# Change in Rainfall Drives Malaria Re-Emergence in Anhui Province, China

**DOI:** 10.1371/journal.pone.0043686

**Published:** 2012-08-21

**Authors:** Hong-Wei Gao, Li-Ping Wang, Song Liang, Yong-Xiao Liu, Shi-Lu Tong, Jian-Jun Wang, Ya-Pin Li, Xiao-Feng Wang, Hong Yang, Jia-Qi Ma, Li-Qun Fang, Wu-Chun Cao

**Affiliations:** 1 State Key Laboratory of Pathogen and Biosecurity, Beijing Institute of Microbiology and Epidemiology, Beijing, People’s Republic of China; 2 National Center for Public Health Surveillance and Information Service, Chinese Centre for Disease Control and Prevention, Beijing, People’s Republic of China; 3 Environmental and Global Health, College of Public Health and Health Professions, and Emerging Pathogens Institute, University of Florida, Gainesville, Florida, United States of America; 4 Anhui Centre for Disease Control and Prevention, Hefei, People’s Republic of China; 5 School of Public Health, Queensland University of Technology, Queensland, Australia; Swiss Tropical & Public Health Institute, Switzerland

## Abstract

Malaria is re-emerging in Anhui Province, China after a decade long’ low level of endemicity. The number of human cases has increased rapidly since 2000 and reached its peak in 2006. That year, the malaria cases accounted for 54.5% of total cases in mainland China. However, the spatial and temporal patterns of human cases and factors underlying the re-emergence remain unclear. We established a database containing 20 years’ (1990–2009) records of monthly reported malaria cases and meteorological parameters. Spearman correlations were used to assess the crude association between malaria incidence and meteorological variables, and a polynomial distributed lag (PDL) time-series regression was performed to examine contribution of meteorological factors to malaria transmission in three geographic regions (northern, mid and southern Anhui Province), respectively. Then, a two-year (2008–2009) prediction was performed to validate the PDL model that was created by using the data collected from 1990 to 2007. We found that malaria incidence decreased in Anhui Province in 1990s. However, the incidence has dramatically increased in the north since 2000, while the transmission has remained at a relatively low level in the mid and south. Spearman correlation analyses showed that the monthly incidences of malaria were significantly associated with temperature, rainfall, relative humidity, and the multivariate El Niño/Southern Oscillation index with lags of 0–2 months in all three regions. The PDL model revealed that only rainfall with a 1–2 month lag was significantly associated with malaria incidence in all three regions. The model validation showed a high accuracy for the prediction of monthly incidence over a 2-year predictive period. Malaria epidemics showed a high spatial heterogeneity in Anhui Province during the 1990–2009 study periods. The change in rainfall drives the reemergence of malaria in the northern Anhui Province.

## Introduction

Malaria, a highly climate-sensitive vector-borne infectious disease, causes approximately 250 million clinical episodes and 1 million deaths annually in more than 92 countries worldwide [1]. Despite a significant decrease in malaria incidence in China at the end of the 20^th^ century, the disease still presents an important public health problem.

Historically, malaria was a serious health threat in Anhui Province of eastern China. In 1980, the number of malaria cases in Anhui Province reached 1.12 million, accounting for 33.9% of the malaria cases reported in the entire country. The Central Government paid great attention to anti-malarial programs, including programs focused on policy planning and analysis, funding the allocations and development of technical guidelines for malaria control and prevention. Several regulations were designed by the Ministry of Health, including the “National Program of Malaria Control in China”, “Management Measures of Malaria Control”, and “Technical Strategies for Malaria Control” [2]. The number of cases decreased dramatically and endemic areas of malaria diminished through the active implementation of the Malaria Control Program. In the late 1990s, many counties in Anhui Province had met the criteria for the basic elimination of malaria (i.e., the incidence was less than 1/10,000 population) [2]. The annual incidence of malaria in the entire province was reduced from 52.0 cases per 100,000 population in 1990 to 1.3 cases per 100,000 population in 1999. By 2000, malaria had reemerged in the region along the Huanghuai River Basin in Anhui, Henan and Jiangsu Provinces located in central China [3]–[6]. In Anhui Province, both the number of reported cases and the incidence of malaria were the highest in the country, with 34,984 malaria cases reported in 2006. This accounted for 54.5% of the total number of reported cases in mainland China [7].

The resurgence of malaria in Anhui Province has aroused the great concern of various levels of governments and also members of academia. Every effort has been made in controlling the disease. However, the factors contributing to its re-emergence remain unclear. Malaria transmission is known to be affected by various factors. It is widely accepted that climatic conditions have a significant impact on malaria transmission by influencing the abundance of *Anopheles* mosquitoes, the frequency with which they bite, and the extrinsic incubation period (EIP) of the parasites within mosquitoes [8]–[13]. However, the association between global warming and the worldwide increase in malaria incidence is still disputed [12], [14]–[23]. There is much uncertainty about the potential impact of climatic factors on malaria at both local and global scales, and the topic remains of considerable interest to researchers [18]. In addition, previous studies have mostly involved malaria that was caused by *Plasmodium falciparum*. The impacts of climatic factors on malaria caused by *P. vivax*, which has different vectors and characteristics of transmission from *P. falciparum*, warrants investigation.

Anhui Province is a *vivax* malaria endemic area where the primary vector responsible for transmission of the disease is *Anopheles sinensis* which develops in bodies of water on the paddy field, gully and the small streams near human residences. The general approach in the study area for controlling *vivax* malaria is to detect and cure patients as early as possible in order to eliminate infectious sources and to control mosquito in and around the residences [2]. Thus, it is essential to understand the spatial and temporal distribution of reported cases and to identify the hotspots of malarial incidence to target interventions. In this study, we aim to describe the distribution of malaria cases over time and space and to explore the potential effects of climatic variables on malaria transmission across different regions in Anhui Province, using long-term historical surveillance data collected from 1990 to 2009.

## Results

**Figure 1:**
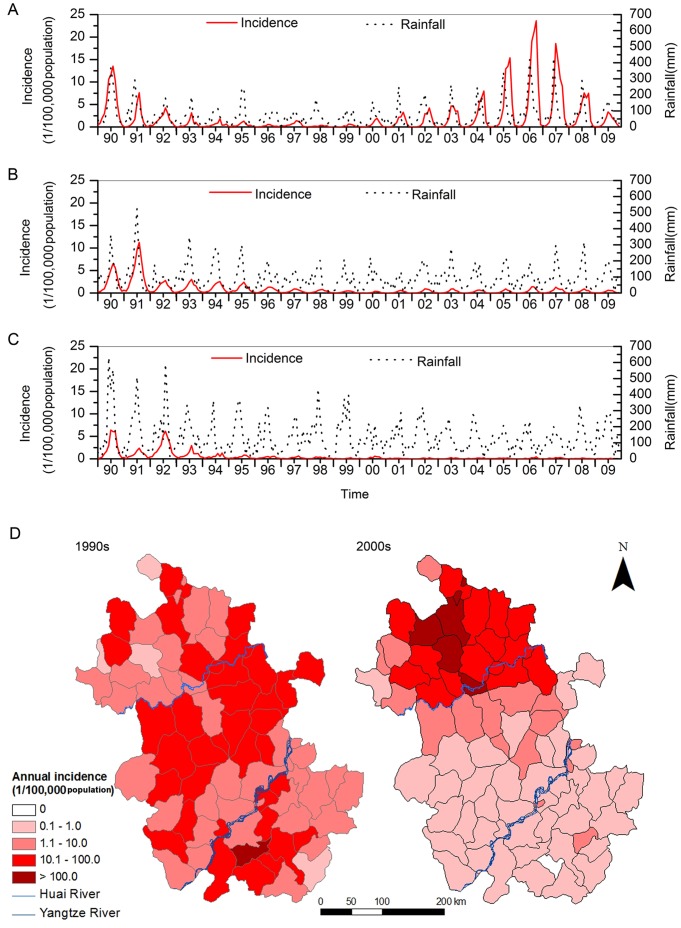
Spatial and temporal distribution of malaria in Anhui Province, China from 1990 to 2009. (A) Monthly malaria incidence and rainfall in northern Anhui Province; (B) Monthly malaria incidence and rainfall in mid Anhui Province; (C) Monthly malaria incidence and rainfall in southern Anhui Province; (D) Average annual malaria incidence at the county level in Anhui Province in the 1990s and 2000s.

A total of 198,875 cases of malaria were reported from 1990 to 2009, distributed in all 78 counties of Anhui Province. The annual incidence varied from 52.01 cases per 100,000 population in 1990 to 11.9 cases per 100,000 population in 2009, with a median of 10.7 cases per 100,000 population in Anhui Province. The spatial variation in malaria incidences over the province’s counties showed that the annual average incidence ranged from 0.45 to 88.65 cases per 100,000 population with a median of 10.45 cases per 100,000 population from 1990 to 2009. The cumulative number of malaria cases in each county from 1990 to 2009 ranged from 40 to 22,262 with a median of 955. Malaria incidence significantly decreased in all three geographic regions during the 1990s. The annual incidence declined from 64.89 to 1.77 cases per 100,000 population in the northern Anhui Province, from 49.43 to 2.48 cases per 100,000 population in the middle of the province, and from 30.76 to 0.73 cases per 100,000 population in the southern Anhui Province. After 2000, a rapid increase in malaria incidence was reported. The annual incidence peaked in 2006, reaching 90.54 cases per 100,000 population in the northern Anhui Province (Figure 1-A). Nevertheless, the annual incidences remained at relatively low levels in the other two geographic regions, with a mean of 3.47±1.29 cases per 100,000 population, and 1.09±0.54 cases per 100,000 population in the mid and southern Anhui Province from 2000 to 2009, respectively (Figure 1-B, C). The hotspots of malaria shifted over the past two decades, from counties in all three geographic regions in the 1990s to counties in the northern Anhui Province in the 2000s (Figure 1-D).

Spearman correlation analyses showed that the monthly incidence of malaria was significantly associated with some climatic variables, including temperature, relative humidity (RH), rainfall and The Multivariate ENSO Index (MEI) in all three geographic regions, with varying lag effects ranging from 0 to 2 months (Table 1). Rainfall was found to have the highest correlation with the incidence of malaria (r_s_≥0.48), followed by RH, MEI, and temperature.

**Table 1:** Spearman correlation coefficient (95% confidence intervals) between the monthly incidence of malaria and climate variables in Anhui Province, 1990–2009.

Region	MaxT	T	MinT	RH	Rainfall	MEI
Northern	L1 = 0.33	L1 = 0.32	L1 = 0.31	L0 = 0.33	L1 = 0.55	L1 = 0.19
	(0.22 to 0.42)	(0.21 to 0.42)	(0.20 to 0.41)	(0.20 to 0.45)	(0.47 to 0.64)	(0.07 to 0.33)
Mid	L1 = 0.23	L1 = 0.22	L1 = 0.21	L0 = 0.34	L2 = 0.53	L1 = 0.35
	(0.11 to 0.35)	(0.10 to 0.34)	(0.10 to 0.31)	(0.26 to 0.45)	(0.44 to 0.62)	(0.23 to 0.47)
Southern	L1 = 0.32	L1 = 0.32	L1 = 0.31	L1 = 0.23	L2 = 0.48	L1 = 0.39
	(0.19 to 0.44)	(0.20 to 0.44)	(0.21 to 0.42)	(0.08 to 0.33)	(0.37 to 0.59)	(0.28 to 0.49)

Lx: the lagged months. MaxT: average maximum temperature; T: average temperature; MinT: average minimum temperature; RH: relative humidity; MEI: multivariate El Niño/Southern Oscillation index.

**Figure 2:**
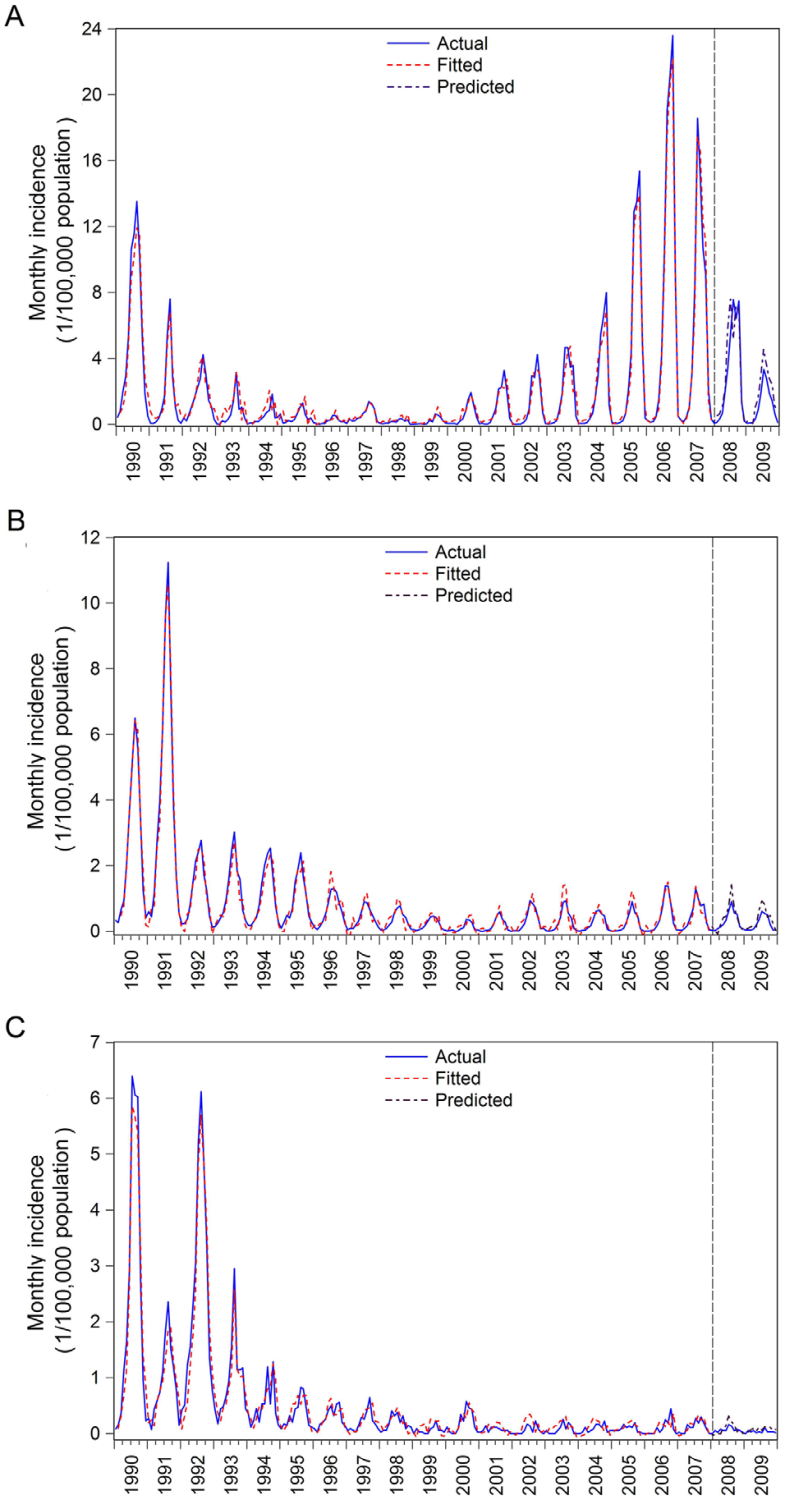
Validations of polynomial distributed lag models of malaria incidence in three regions, Anhui Province, China. The data from January 1990 to December 2007 were used to construct the models, and the data from January 2008 to December 2009 were used for the validation of the models. (A) In northern Anhui Province; (B) In mid Anhui Province; (C) In southern Anhui Province.

The PDL time-series regression analyses revealed that only rainfall with lags of 1–2 months was significantly associated with the malaria incidence in all three regions. The model yielded the best fit according to the the root mean square error (RMSE) (Model I) in the northern Anhui Province, in which rainfall was significantly associated with malaria incidence at the quadratic term (β = −1.845, p = 0.008) and the cubic term (β = 1.245, p = 0.017) (Table 2). Model II showed that RH was also significantly associated with malaria incidence at the quadratic term (β = 0.764, p = 0.004) and the cubic term (β = −0.085, p = 0.051)_._ Model III showed that RH was not significantly associated with malaria incidence after adjusting for rainfall. Average temperature, maximum and minimum temperature, and MEI were not significantly associated with malaria incidence and were therefore excluded in these models. Rainfall was also significantly associated with malaria incidence in both the mid and southern Anhui Province (Table 3). The PDL models showed that rainfall at the 1–2 month lag was positively associated with monthly malaria incidence in all three regions (Table 4). The validation of these PDL models using data from 2008 to 2009 demonstrated a good fit between observations and predictions, and the high predictive powers of these models were achieved using the two-year observations in all three regions (Figure 2).

**Table 2:** Polynomial distributed lag time-series regression coefficients of the monthly rainfall and relative humidity associated with malaria incidence in northern Anhui Province, 1990–2009^a^.

Variables	Model I	Model II	Model III		
	Rainfall (100 mm)	Relative humidity (10%)	Rainfall (100 mm)		Relative humidity (10%)
	β (95% CI)	β (95% CI)	β (95% CI)		β (95% CI)
Constant term	2.485*	−0.894**	1.473**		0.093
	(0.285 to 4.686)	(−0.939 to −0. 848)	(0.471 to 2.476)		(−0.753 to 0.939)
Linear coefficient	−2.696	−0.251	−1.152		−0.044
	(−5.542 to 0.150)	(−1.957 to 1.455)	(−2.206 to −0.097)		(−1.066 to 0.978)
Quadratic coefficient	−1.845**	0.764**	−1.672**		0.063
	(−3.191 to −0.499)	(0.243 to 1.285)	(−2.902 to −0.441)		(−1.015 to 1.142)
Cubic coefficient	1.245*	−0.085	0.960*		−0.116
	(0.231 to 2.260)	(−0.231 to 0.062)	(0.193 to 1.727)		(−0.773 to 0.541)
R-square	0.92	0.89		0.95	
AIC	7.07	8.33		5.98	
RMS error	1.56	2.82		1.63	

a Adjusting for auto-correlation; no significant association was found with temperature and MEI (p>0.10).

** p<0.01,

* p<0.05.

**Table 3:** Polynomial distributed lag time-series regression coefficients of the monthly rainfall associated with malaria incidence in mid and southern Anhui Province, 1990–2009^a^.

Variables	Mid Rainfall(100 mm)		Southern Rainfall(100 mm)	
	β	95% CI	β	95% CI
Constant term	0.379**	(0.256 to 0.502)	1.261*	(0.200 to 2.323)
Linear coefficient	−0.080	(−0.182 to 0.021)	0.570*	(0.094 to 1.046)
Quadratic coefficient	−1.536**	(−1.608 to −1.465)	−0.477**	(−0. 818 to −0.135)
Cubic coefficient	2.263**	(2.005 to 2.522)	–	–
R-square	0.93		0.90	
AIC	1.40		6.75	
RMS error	1.86		1.75	

a Adjusting for auto-correlation; no significant association was found with temperature, humidity and MEI (p>0.10).

** p<0.01.

* p<0.05.

**Table 4:** Lag distribution coefficients of the monthly rainfall (100 mm) with malaria incidence in the three regions of Anhui Province, China during 1990–2009.

Variables	Northern		Mid		Southern		
	β	95% CI	β	95% CI	β	95% CI	
Lag0	0.234	(−0.448 to 0.915)(0.492 to 1.862)(−1.173 to 0.215)(−0.741 to 0.618)	0.203	(−1.349 to 1.756)(0.712 to 2.310)(−1.143 to 1.659)(−0.683 to 1.808)	0.205	(−0.609 to 1.018)(0.102 to 2.363)(0.255 to 2.504) (−0.327 to 1.338)	
Lag1	1.177 **		1.511**		1.232**		
Lag2	−0.479		0.258		1.125 *		
Lag3	−0.061		0.562		0.832		

** p<0.01.

* p<0.05.

## Discussion

Malaria is reemerging in Anhui Province of China. The number of human malaria cases has increased rapidly since 2000 after a low level of endemicity at the end of 1990s. Using long-term surveillance data from 1990 to 2009, this study described the spatial and temporal patterns of malaria epidemics at a regional level and explored the potential effect of climatic variables on malaria incidence in a region of Anhui Province that is highly endemic in malaria and has experienced a resurgence of malaria incidence. We observed substantial spatial heterogeneity in the incidence of malaria in different regions of Anhui Province, and found rainfall to be the most important meteorological factor for the reemergence of malaria in Anhui Province. The modeling results show that 92%, 93% and 90% of the variance in malaria transmission was accounted for by rainfall in the northern, middle and southern Anhui Province, respectively. The PDL model was used based on biological consideration because rainfall can affect malaria transmission not only in the same month as the rainfall occurs, but also in several continuous months. The overall effect of an increase in rainfall is its impact on malaria transmission in that month plus its impact on continuous months. The high predictive powers for the prediction of monthly incidences of malaria, combined with the effect of rainfall with lags of 1–2 months on malaria incidence reported in this study, provide a good justification for using the PDL models to make short-term prediction regarding malaria epidemics in the resurgent areas. The predictive powers are also helpful for mobilizing the limited public health resources more effectively and efficiently.

In the early 1990s, the incidence of malaria was high throughout the entire Anhui Province. The incidence then significantly decreased. However, the reemergence of malaria has become a major public health problem in the northern areas of Anhui Province since 2000. In the meantime, malaria transmission remained at relatively low levels in the mid and southern Anhui Province. The reasons that malaria resurgence occurred only in the northern Anhui Province and not in other regions of Anhui Province are complex. Since 2000, social-economic activities in Anhui have greatly changed. Many factors, such as the promotion of the development of urbanization and the constructions of national highways and expressways mainly focused on the northern region. In this region, social-economic development remained relatively stagnant, and there was an acceleration in the movement of non-immune migrants such as travellers and labourers. These factors have facilitated malaria transmission patterns. Frequent population movement exacerbates the malaria transmission problem in Anhui, and identifying and understanding the influence of human population movement can improve malaria prevention and control measures [24]. On the other hand, resistance of *P. vivax* to the commonly used drugs (chloroquine and primaquine which are used in malaria treatment) during these years may have contributed to the total malaria case occurrence [25]. Malaria transmission is determined by various factors, such as population immunity, vector species and abundance, mosquito control measures, social and economic status and environment factors [26]–[30]. However, the climate provides the framework for transmission of malaria, and other factors can only affect malaria transmission in spatiotemporal zones where the climate is suitable [23]. Meteorological factors including temperature, relative humidity, and rainfall affect the developmental periods of different stages in malaria transmission. PDL model methods were applied to quantify the seasonal pattern variation of *P. vivax* and the effects of the climate, and these results indicated the important roles of rainfall in driving seasonal patterns of malaria incidence, implying that malaria transmission was more sensitive to rainfall in this study area. Other studies in Africa have also suggested that rainfall, rather than temperature, was the primary driving force of *P. falciparum* malaria transmission [31], [32], and a positive correlation of rainfall with malaria incidence was also reported in India [33]. However, studies in areas of south Asia reported no significant relationship, or only a weak association, between rainfall and malaria incidence [34], [35]. There is no clear explanation for such heterogeneity of the results of the different studies, but difference could be due to different local climates or the types of local mosquito vectors.

*P. vivax* is the predominant species of causative agent of malaria and *A. sinensis* is the major vector in the study area [5], which develops in accumulated bodies of water such as the paddy field, gullies and small stream. It is biologically plausible that *A. sinensis* abundance often increases with moderate rainfalls and that susceptible people become sick easily after being bitten by mosquitoes. The rainfall may directly affect the mosquito density, which then affects malaria transmission, given that other socio-ecological factors remain constant [36]. Better entomological data would greatly increase our understanding of the influence of rainfall on the biology of malaria [37]. Unfortunately, such entomological data are not yet available in our study areas. Climatic variables are considered as the environmental factors for increased risk of malaria because of their impacts on both the *Plasmodium* incubation rate and mosquito vector activities. To understand the contribution of climatic factors to the transmission of malaria in Anhui Province, a PDL model between malaria incidence and climatic variables was conducted. Among the climatic factors investigated in the study, rainfall was the most influencing factor, especially with a 1∼2 month lag effect. It is known that moderate rainfall provides additional breeding sites for the aquatic stages of the mosquito life cycle and also increases the relative humidity. Moderate temperate and relative humidity affect the longevity of the adult mosquito so that it can transmit the infection for a longer period of time. Time-series regression analyses suggest that the impact of rainfall on malaria follows a lag of 1–2 months, and such delays are consistent with the generation time of a case of *P. vivax* (vector-parasite-host cycle under optimum conditions) in this study area. Generating a case of *P. vivax* usually requires 11–25 days including approximately eight days in the exoerythrocytic stage, two days in the erythrocytic stage, and an extrinsic incubation period of about nine days in the body of the mosquito [38]. However, the relationship between mosquito abundance and rainfall was nonlinear. Moderate rainfall could provide beneficial breeding grounds such as the formation of small puddles, and the growth of mosquitoes helps the parasite complete the life cycle, allowing for increased transmission of the parasite. However, it should be noted that excessive rainfall might flush out the mosquito breeding sites and destroy the larvae, varying with greatly over time and in space [39]. In addition to meteorological factors, the seasonal variation of malaria transmission or vector abundance may be influenced by many other factors such as the fluctuations of migrant workers and socioeconomic factors such as ethnic group, parents’ education and occupation, use of protective measures and the family’s financial status [32]. Further studies are required to explore the effect of these variables on malaria transmission in the region.

The limitations of this study should be acknowledged. First, the data were collected from a passive surveillance system, and under-reporting bias is inevitable. For example, some people with mild clinical symptoms, self-treated cases or people with non-apparent clinical infection might not seek any medical attention. This would lead to an underestimation of the extent of malaria epidemics and could impact the precision of the models. However, it seems unlikely that disease severity would have an inter-annual component that would influence the general pattern of the observed results [40]. Second, malaria incidence is determined not only by climatic variability but also by other factors such as population immunity, mosquito control measures, and social and economic status. However, these data were unavailable for this study, which covers such an extended period of time.

In summary, there was a significant association between malaria transmission and rainfall in Anhui Province. Therefore, the results of this study may facilitate the development of early warning systems and support intervention measures for reducing the incidence of malaria in this region and in nearby areas displaying a reemergence in malaria cases.

## Materials and Methods

### Study Area

**Figure 3:**
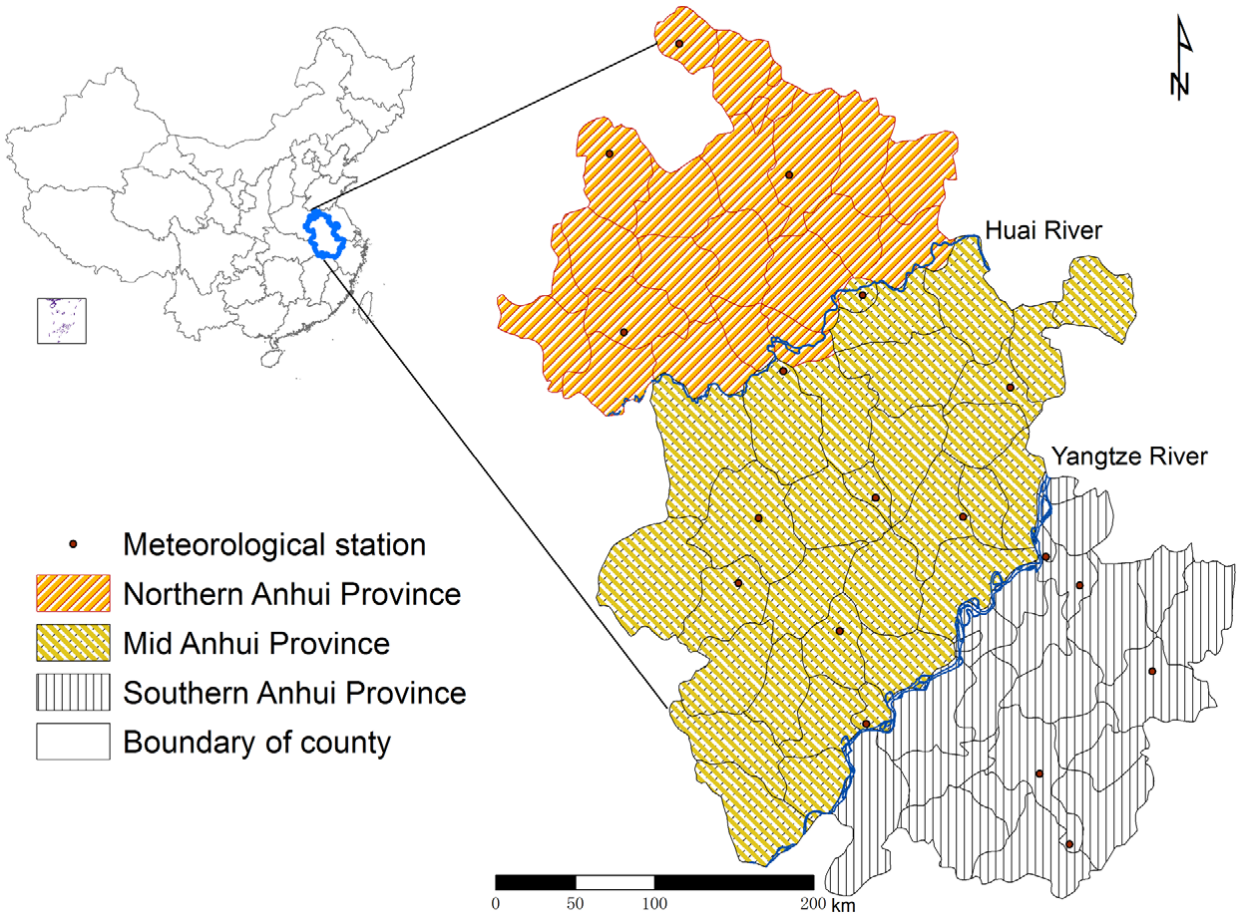
Study areas in Anhui Province, China. Northern Anhui Province includes 22 counties in the north of the Huai River. Mid Anhui Province includes 32 counties between the Huai River and the Yangtze River. Southern Anhui Province includes 24 counties in the south of the Yangtze River.

The study area covers Anhui Province, an inland province in eastern China, that is located between 29°38′∼34°74′ north, and 114°85′∼119°69′ east and has a total land area of 139,600 square kilometers and a population of approximately 67 millions. The largest (the Yangtze) and third largest (the Huai) rivers in China run through the province, from west to east, dividing the province into 3 geographic regions, i.e., northern, mid and southern regions. The northern region has 22 counties in the north of the Huai River. The mid region is located between the Huai River and the Yangtze River and includes 32 counties. The southern region consists of 24 counties located south of the Yangtze River (Figure 3). The northern region of Anhui Province, as part of the North China Plain, is a vast expanse of flatland. The mid region includes a chain of undulating hills as well as the low and flat lands along the Yangtze River and around the Caohu Lake. The southern region is mainly covered by hills. Anhui Province is located between the warm-temperate zone and subtropical zone, and the weather is warm and humid in the summer and has four distinct seasons. Seasonal rainfall varies greatly, usually with dry springs and wet summers, and the annual average rainfall ranges from 800 to 1,800 millimeters. The annual average temperature is between 14 and 17 degrees centigrade.

### Data Collection and Management

The China Ministry of Health lists malaria as a notifiable disease and maintains routine surveillance of clinical malaria cases nationwide. The physicians working at medical institutions and primary health care facilities including those in the private sector, in both urban communities and rural areas are required to report all diagnosed malaria cases to the Centre for Disease Control and Prevention at the county level, and are also required to report to the higher Centre for Disease Control and Prevention. The monthly reported cases from 1990 to 2009 for each county were new cases and were obtained from the Anhui Centre for Disease Control and Prevention. The disease is diagnosed according to the guidelines of the World Health Organization (WHO). Briefly, the two recommended methods, optic microscopy and rapid diagnostic tests based on lateral flow immunochromatography, support the clinical management of malaria [41]. The census data of each county were obtained from the Anhui Provincial Bureau of Statistics. The annual incidence of malaria for each county was geo-referenced and matched to the corresponding county on a digital map of Anhui Province using ArcGIS9.2 (ESRI Inc., Redlands, CA).

The monthly meteorological data for the same time period, including average minimum temperature (MinT), average temperature (T), average maximum temperature (MaxT), relative humidity (RH) and rainfall, were extracted from 18 national meteorological monitoring stations in the China Meteorological Data Sharing Service System (Figure 3) [42]. The China National Meteorological Information Centre processes and publishes the monthly *in situ* meteorological observations for each province, region and municipality according to a uniform protocol. In addition, El Niño/Southern Oscillation (ENSO) is the most important coupled oceanic-atmospheric phenomenon that affects climate variability in many regions of the world, in particular Pacific Ocean regions. Accumulating evidence has shown an association between ENSO and malaria epidemics in parts of southern Asia and South America [43], [44]. The Multivariate ENSO Index (MEI), developed to monitor ENSO, is an integrated quantity based on the six main observed variables in the tropic Pacific including sea-level pressure, zonal and meridional components of the surface wind, sea surface temperature, surface air temperature, and the total cloudiness fraction of the sky. This index is available from the Earth System Research Laboratory of the National Oceanic and Atmospheric Administration [45].

### Spatial and Temporal Analysis of Malaria Incidence

The monthly malaria incidence was calculated for each county, and the epidemic curves for three geographic regions were plotted to explore the temporal pattern of malaria transmission in Anhui Province from 1990 to 2009. To explore the spatial pattern of malaria transmission, the average annual malaria incidences in each county during the 1990s and 2000s were separately mapped.

### Analyses of the Effect of Climatic Factors on Malaria Transmission

Spearman correlation analyses were conducted to examine the association between the monthly malaria incidence and each of the climatic variables for the three geographic regions. Lags of months for climatic variables were used in the analysis to explore the lagged effects. To examine the contribution of climatic factors to malaria transmission, a polynomial distributed lag (PDL) time-series regression was performed by taking the autocorrelation of the lagged effects into account [46]. The PDL model was constructed as follows: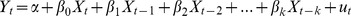 where the coefficients 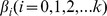 describe the lagged effects of 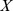
on
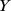
and 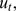 which represent the residual.

Letting 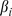 represent a polynomial of degree *m* in *i* :
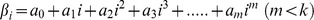


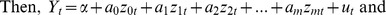


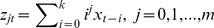



In this study, the monthly incidence of malaria was used as a dependent variable, and meteorological variables (MaxT, T, MinT, RH, rainfall, and MEI) were used as the independent variables. Akaike’s information criterion (AIC) was used to measure goodness-of-fit of the PDL models. The predictive validity of the models was evaluated using the root mean square error 
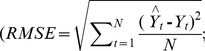
 where 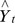 is the predicted value for month *t*, 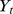 is the observed value, and *N* is the number of observations). The data spanning the period of January 1990 to December 2007 were used to construct and optimize the models, while the data from January 2008 to December 2009 were used to assess the predictive ability of these models.
